# Changes in Serum Neutralizing Antibodies Levels During Convalescence of COVID-19 Patients

**DOI:** 10.3389/fmed.2022.829273

**Published:** 2022-02-11

**Authors:** Qing-Qing Chen, Lei Gong, Xiao-Min Wu, Ya-Ting Feng, Wan-Rong Luo, Xue Zhou, Yuan Yuan, Jun-Ling Yu, Lan He, Peng Wang, Ying-Lu Ge, Sai Hou, Wei-Wei Li, Yong Sun, Jia-Bing Wu, Bin Su, Hai-Feng Pan, Jun He, Zhi-Rong Liu

**Affiliations:** ^1^Microbiological Laboratory, Anhui Provincial Center for Disease Control and Prevention, Hefei, China; ^2^Microbiological Laboratory, Public Health Research Institute of Anhui Province, Hefei, China; ^3^Department of Epidemiology and Biostatistics, School of Public Health, Anhui Medical University, Hefei, China; ^4^Inflammation and Immune Mediated Diseases Laboratory of Anhui Province, Hefei, China; ^5^School of Public Health, Anhui Medical University, Hefei, China

**Keywords:** COVID-19, antibody, microneutralization, cohort study, vaccination

## Abstract

Detection of serum-specific SARS-CoV-2 antibody has become a complementary means for the identification of coronavirus disease 2019 (COVID-19). As we already know, the neutralizing antibody titers in patients with COVID-19 decrease during the course of time after convalescence, whereas the duration of antibody responses in the convalescent patients has not been defined clearly. In the current study, we collected 148 serum samples from 37 confirmed COVID-19 cases with different disease severities. The neutralizing antibodies (Nabs), IgM and IgG against COVID-19 were determined by CLIA Microparticle and microneutralization assay, respectively. The time duration of serum titers of SARS-CoV-2 antibodies were recorded. Our results indicate that IgG (94.44%) and Nabs (89.19%) can be detected at low levels within 190–266 days of disease onset. The findings can advance knowledge regarding the antibody detection results for COVID-19 patients and provide a method for evaluating the immune response after vaccination.

## Introduction

Coronavirus disease 2019 (COVID-19) is an emerging global infectious disease caused by the severe acute respiratory syndrome coronavirus 2 (SARS-CoV-2) (1). As of August 2021, according to the weekly epidemiological update on COVID-19 issued by World Health Organization, the cumulative number of confirmed COVID-19 cases worldwide has exceeded 200 million in over 200 countries, just 6 months after reaching 100 million cases.

The SARS-CoV-2 genome encodes four structural proteins including spike (S), nucleocapsid (N), envelope, and membrane proteins. The receptor-binding domains (RBD) in the S1 region of S protein are the main target of neutralizing antibodies (Nabs) and can be a potential target for vaccine development. The presence of Nabs is one of the most important indicators of clinical outcome and vaccination effectiveness in other respiratory viral infections. So far, the COVID-19 vaccine has been widely used, but it is still not very clear how much protection people will get after being vaccinated. The assessment of the immune protection status through testing the specific Nabs is particularly important. Nabs are important for viral clearance and are considered key to recovery and protection against viral diseases. Nabs can reduce viral infectivity by binding to the surface epitopes of viral particles and thereby blocking the entry of the virus to an infected cell. Nabs elicit their protective activities in three main steps and it may prevent the attachment of the virion to its receptors on targeted cells, causing aggregation of virus particles. Analysis of the relationship between SARS-CoV-2 viral load and dynamics profiles of antibodies responses is scarce. It has been suggested that the detection of antibodies to SARS-CoV-2 could serve as the basis for an “immunity passport,” but it is currently unclear whether recovered COVID-19 cases have Nabs that protect them from a second infection. Currently, virus neutralization assays are considered as gold standard serological methods. Therefore, in the present study, we used the microneutralization test to study the dynamic changes of Nabs in COVID-19 patients, aiming to provide evidence for evaluating the efficacy of the vaccine.

## Materials and Methods

### Subjects

A total of 37 laboratory confirmed convalescent COVID-19 patients (20 female and 17 male), aged between 14 and 78 years (median age: 48 years), were recruited in Anhui Province of China from January to February 2020. A total of 35 respiratory specimens were obtained from 26 patients, including 19 throat swabs and 16 sputum samples. A total of 148 sera were collected from the 37 patients based on varying disease course. According to disease classifications outlined in the “Guidelines on the Novel Coronavirus-Infected Pneumonia Diagnosis and Treatment (Eighth Edition)” issued by the National Health Commission of China (NHC), 4 (10.81%) patients with mild symptoms, 23 (62.16%) patients with moderate symptoms, and 10 (27.03) patients with severe symptoms were enrolled. Respiratory specimens were detected during acute phases of infection (0–14 d, median: 3.5 d). The serum samples were collected from all patients during visits at four different follow-up time points: the acute/early specimen (0–14 d, median: 4 d), the convalescent/late specimen visit 1 (22–98 d, median: 77 d), visit 2 (104–148 d, median: 121 d), and visit 3 (190–266 d, median: 214 d; Table 1).

**Table 1:** The demographic characteristics and four different following time points of follow-up patients.

**Characteristic**	**Participants**			
	**Mild symptoms (*n* = 4)**	**Common symptoms (*n* = 23)**	**Severe symptoms (*n* = 10)**	**Overall (*n* = 37)**
Age, median (range), years	16 (14–41)	43 (22–69)	57.5 (40–78)	48 (14–78)
**Sex, no. (%)**				
Male	2 (50.00)	9 (39.13)	6 (60.00)	17 (45.94)
Female	2 (50.00)	14 (60.87)	4 (40.00)	20 (54.06)
**Duration of follow-up, median (range), days**				
Acute phase	3 (0–4)	4 (0–14)	4 (0–10)	4 (0–14)
Visit 1	49 (27–86)	77 (22–98)	78 (29–94)	77 (22–98)
Visit 2	112 (109–115)	121 (104–143)	130 (112–148)	121 (104–148)
Visit 3	205 (201–206)	213.5 (190–261)	248 (210–266)	214 (190–266)

### Serological Tests

After the sera were inactivated at 56°C for 30 min, the IgM and IgG antibodies against SARS-CoV-2 were detected using SARS-CoV-2 IgM CLIA Microparticle (MCLIA,CMU0202, Zhengzhou China) and SARS-CoV-2 IgG CLIA Microparticle (MCLIA, CMU0102, Zhengzhou China). Virus Nab titers were evaluated using the microneutralization assay described previously (2). All sera were tested in duplicates in a two-fold dilution series with Dulbecco's Modified Eagle Medium (Gibco, CA, USA) containing 2% fetal bovine serum (Gibco, CA, USA) from 1:16, and then were incubated with 100T CID_50_ virus at 37°C for 1 h. Subsequently, Vero cells (1–2 × 10^4^ per well) were added. Virus-specific cytopathic effects (CPE) were observed on day 5 post-infection. Antibody titers ≥16 indicated a positive result in this study. In this study, the sequence of the virus used in microneutralization assay is accessible from GISAID's database under accession numbers EPI_ISL_413485.

### Real-Time RT-PCR

Gene open reading frame 1ab (ORF1ab) and nucleocapsid protein (N) of SARS-Cov-2 were identified by specific fluorescence quantitative RT-PCR assays, which used commercial kits (BioGerm, 20203400065) qualified by Chinese FDA.

### Statistical Analysis

The correlation analysis was calculated by the Pearson correlation tests. Statistical differences were calculated by the one-way ANOVA test. Statistical analysis were performed using SPSS Statistics 17.0 (IBM).

## Results

**Figure 1:**
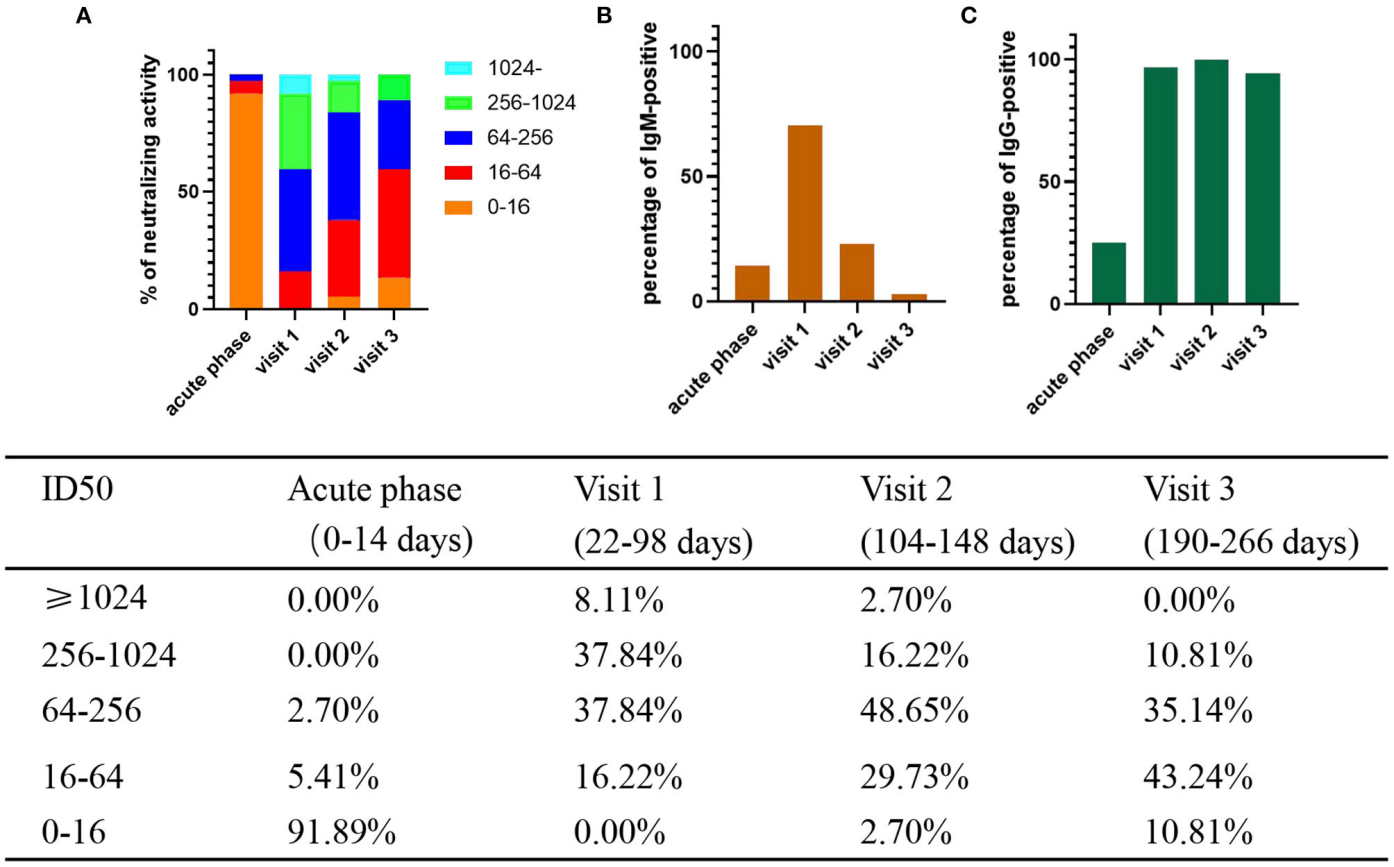
Percentage of levels of neutralizing activity and IgM/IgG-positive and in COVID-19 patients. The positive rate and neutralizing activity was quantified from 37 patients. S/CO ≥1 is positive. **(A)** Different colored boxes depict indicated ID_50_ of SARS-CoV-2 at 4 times after symptom onset **(B)** Percentage of IgM -positive. **(C)** Percentage of IgG -positive.

SARS-CoV-2-specific neutralizing activity was measured for 37 patients by virus neutralization assays. The positive rate of Nabs was 100% (37/37), 97.30% (36/37), and 89.19% (33/37) at visit 1, visit 2, and visit 3, respectively. Then, we compared the changes in the levels of Nabs at each time point. The results showed that the percentage of patients with Nabs titers ≥256 was the highest (17/37, 45.95%) at visit 1, and then declined to 18.92% (7/37) and 10.81% (4/37) at visit 2 and visit 3 (Figure 1A), respectively. The percentage of patients with low titers of Nabs (<64) on the rise from 3 to 9 months increased from 16.22% (6/37) to 32.43% (12/37) and 54.05% (20/37) at visit 2 and visit 3 (Figure 1A), respectively. The neutralizing antibody titers declined at an average of about six-fold from 22–98 to 190–266 days.

In addition, we measured sera IgM and IgG levels of patients. MCLIA tests were positive in 17.24% (5/29) of sera for IgM and 27.59% (8/29) for IgG in acute phase. The percentage of IgM-positive reached peak (71.43%, 25/35) at visit 1, and then decreased to 25.00% (9/36) at visit 2, and to 5.56% (2/36) in visit 3 (Figure 1B). However, the percentage of IgG-positive remained at a high rate: 97.14% (34/35) in visit 1, 100% in visit 2 (36/36), and 94.44% (34/36) in visit 3 (Figure 1C).

**Figure 2:**
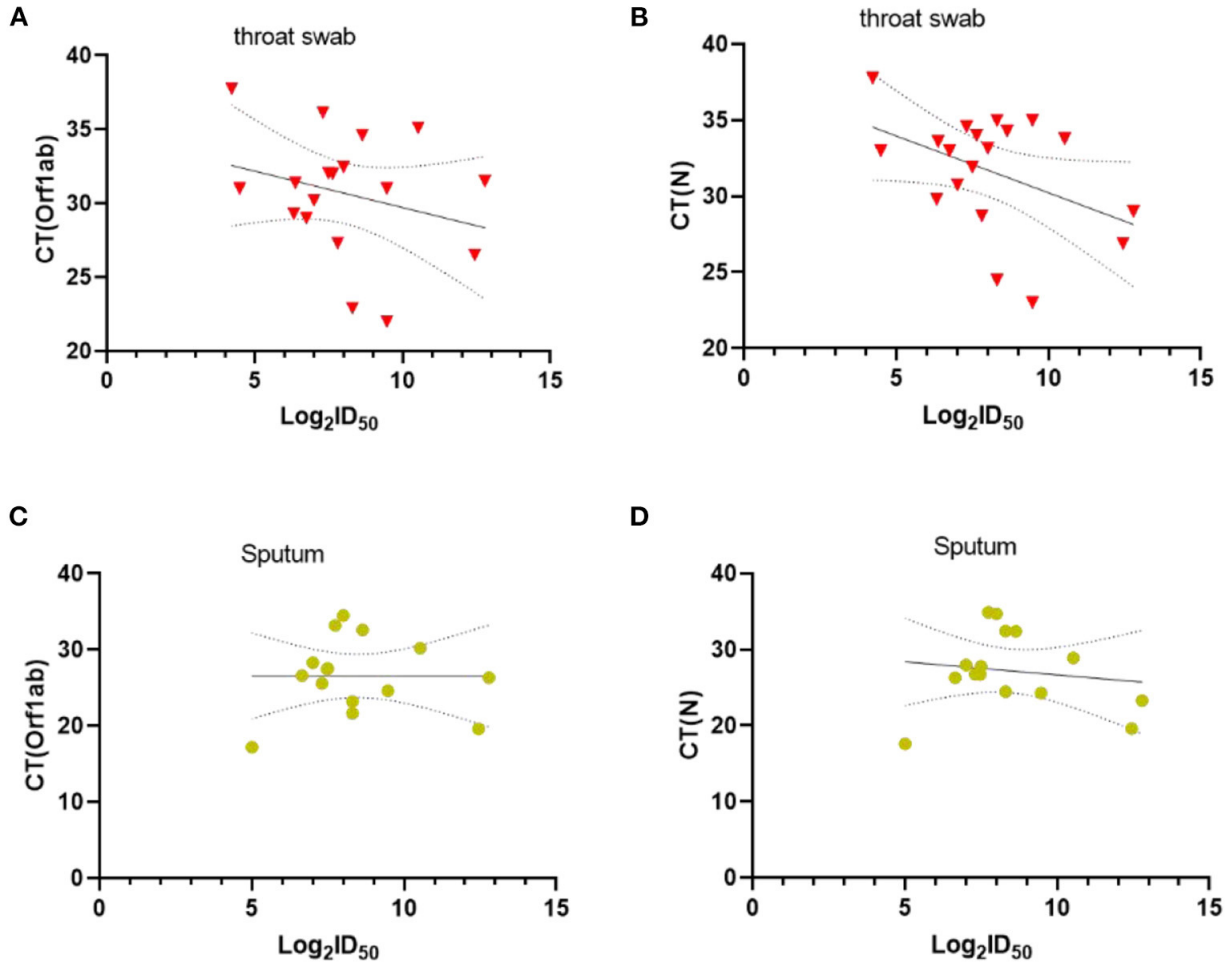
Correlations between CT values of *Orf1ab* and *N* genes and SARS-CoV-2-specific Nabs titer in visit 1, Throat swab **(A,B)** and Sputum **(C,D)**. The dashed area indicates 95% confidence bands of the best-fit line. *P*-values were determined using a Pearson correlation tests.

**Figure 3:**
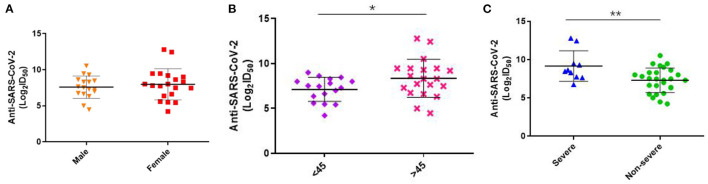
Levels of SARS-CoV-2-specific Nabs titer in different gender, severity and age at visit1. **(A)** Comparison of ID50 between male and female at 1–3 months post-symptom onset (*F* = 0.385, *p* = 0.539). **(B)** Comparison of ID50 between more or <45 age at 1–3 months post-symptom onset (*F* = 4.271, *p* = 0.046). **(C)** Comparison of ID50 between severe and non-severe at 1–3 months post-symptom onset (*F* = 8.617, *p* = 0.006). *P*-values were determined using a one-way ANOVA test. *p < 0.05; **p < 0.01.

Besides, we assessed the level of Nabs in relation to viral loads in throat swabs/sputum, age, sex, and disease severity. SARS-CoV-2-specific genes, Orf1ab and N genes, were measured by RT-PCR. The results showed that there was no correlation between Ct values of Orf1ab and N genes and Nabs titers (visit 1) in throat swab and sputum samples (Figure 2). Stratified analyses were also undertaken to compare neutralizing activities in visit 1 by sex, age, and disease severity. The results showed that both disease severity and age had correlation with Nabs titers (Figures 3B,C), but sex had no correlation with Nabs titers (Figure 3A).

**Figure 4:**
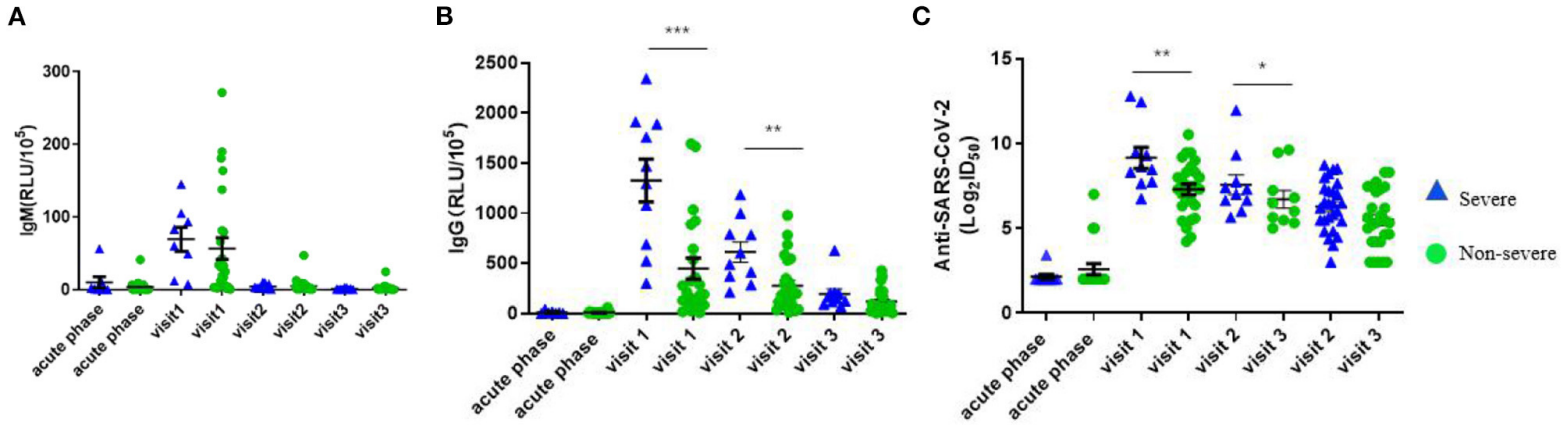
Variation value of RLU (IgM and IgG) and AntiSARS-CoV-2 (Log_2_ID50) over time in different disease severity. **(A)** RLU (IgM). **(B)** RLU (IgG). **(C)** Anti-SARS-CoV-2 (Log_2_ID_50_). *P*-values were determined using a one-way ANOVA test. *p < 0.05; **p < 0.01; ***p < 0.001 (The blue triangle represents severe patients and the green circle represents non-severe patients).

**Figure 5:**
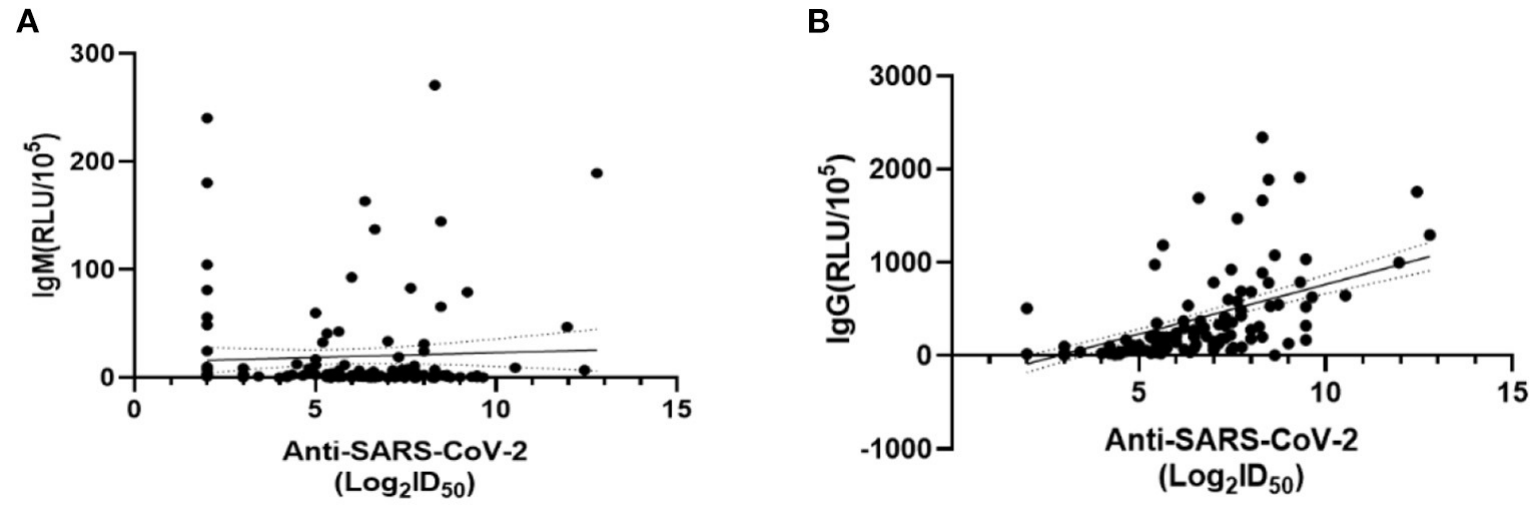
Correlations between SARS-CoV-2-specific Nabs titer and IgM/IgG levels. The dashed area indicates 95% confidence bands of the best-fit line. **(A)** Linear regression analysis of ID_50_ and IgM (RLU/10^5^) (*p* = 0.309, *r* = 0.309). **(B)** Linear regression analysis of ID_50_ and IgG (RLU/10^5^) (*p* < 0.001, *r* = 0.599). *P*-values were determined using a Pearson correlation tests.

We compared the dynamic of IgM/IgG and SARS-CoV-2-specific Nabs titer in the serum of patients with clinical phenotype at each time point. At visit 1, the levels of IgM, IgG, and Nabs peaked. The levels of IgG and Nabs in severe patients were significantly higher than those of non-severe patients at visit 1 and visit 2 (Figures 4A–C). A positive correlation was found between Nabs titers and IgG (Figure 5B, *r* = 0.599, *p* < 0.001), while no significant correlation between Nabs titers and IgM was detected (Figure 5A).

## Discussion

In the current study, 89.19% COVID-19 patients had measurable titers of neutralizing antibody between 190 and 266 days after symptom onset, which was consistent with the findings of recent studies (3, 4). Over 95% COVID-19 convalescents had detectable Nabs and IgG from 6 to 12 months after disease onset (4). A recent study suggested that, Nabs persisted in 89% of subjects at least 13 months after infection (5). Previous studies demonstrated that 83.9% of SARS-CoV patients were positive for Nabs at 36 months after recovery from infection, while the positive PRNT_50_ could be detected 5 years after MERS-CoV (6, 7). A longer longitudinal study needs to be conducted to establish the duration of Nabs. Recently, several studies have reported that neutralizing activity against the Beta and Omicron significantly reduced in serum of COVID-19 convalescent patients infected by the original Strains (3, 8). Although, IgG and Nabs were detectable in recovered SARS-CoV-2 patients at 190–266 days after illness onset, whether the low levels of antibodies have a protective effect on SARS-CoV-2 variants still warrants further exploration.

We further analyzed the factors related to the level of Nabs, and found there was no significant association of neutralizing antibody levels with gender and Ct values of SARS-CoV-2 in throat swabs and sputum. However, a previous study showed a positive correlation between antibodies and mean Ct values in paired nasopharyngeal swabs of COVID-19 patients (9). In a MERS study, the levels of S1 antibodies and Nabs (PRNT_90_ antibody titers) were not correlated with the peak viral loads in sputum, but the duration of the viral shedding was related to the production of Nabs (10). In the current study, Ct value was used as a proxy for assessing SARS-CoV-2 viral load in throat swab and Sputum samples. The variations in specimen collection and nucleic acid extraction/amplification may have an impact on the results of RT-PCR. Several previous studies reported that the levels of Nabs were higher in male patients (11, 12). However, in this study, the sample size may be too small to determine the significance. Besides, our results showed that the disease severity was related to the levels of IgG and neutralizing antibody in early infection of COVID-19, echoing the results of other studies (13–16).

The present study has certain limitations: (1) the sample size was small (37 patients), particularly in severe group; (2) the follow-up period was short (190–266 days); (3) Nabs against SARS-CoV-2 mutant strains were not included; (4) we only investigated antibodies levels but not B-cell immunity and T-cell immunity. Therefore, further in-depth studies with a larger sample size and longer follow-up period are awaited.

COVID-19 has become a major global healthcare challenge, and the spectrum of diseases varies widely. The main challenge of disease management is still to predict severe cases in time to provide active intervention to limit severe disease and subsequent mortality. In summary, our findings describe the dynamics of the serological antibody response to SARS-CoV-2 in COVID-19 patients and the possible influencing factors of Nabs. The findings can advance the knowledge regarding the antibody detection results for COVID-19 patients and provide a method for evaluating the immune response after vaccination.

## Data Availability Statement

The original contributions presented in the study are included in the article/supplementary material, further inquiries can be directed to the corresponding author/s.

## Author Contributions

Z-RL, JH, and H-FP conceived of the presented idea. Q-QC, LG, X-MW, and Y-TF drafted the manuscript and analyzed study data. LG, SH, J-BW, and BS collected specimens. Q-QC, JH, W-RL, XZ, YY, J-LY, LH, PW, Y-LG, W-WL, and YS conducted the experiments. All authors discussed the results and contributed to the final manuscript.

## Funding

This work was supported by the Emergency research project of novel coronavirus infection of Anhui province (202004a07020002), the Scientific Research Projects of Health Commission of Anhui Province in 2021 (AHWJ2021a030), and the opening foundation of the State Key Laboratory for Diagnosis and Treatment of Infectious Diseases, The First Affiliated Hospital, Zhejiang University School of Medicine (SKLID2021KF04).

## Conflict of Interest

The authors declare that the research was conducted in the absence of any commercial or financial relationships that could be construed as a potential conflict of interest.

## Publisher's Note

All claims expressed in this article are solely those of the authors and do not necessarily represent those of their affiliated organizations, or those of the publisher, the editors and the reviewers. Any product that may be evaluated in this article, or claim that may be made by its manufacturer, is not guaranteed or endorsed by the publisher.
